# The Geriatric Population and Psychiatric Medication

**DOI:** 10.4103/0973-1229.58818

**Published:** 2010

**Authors:** Sannidhya Varma, Himanshu Sareen, J.K. Trivedi

**Affiliations:** * *Pursuing Master’s degree in Psychiatry from Department of Psychiatry, CSM Medical University, Lucknow, India.*; ** *Senior Resident, Dept. of Psychiatry, C. S. M. Medical University (Formerly K.G’s Medical University), Lucknow, India.*; *** *Professor, Department of Psychiatry, Chhatrapati Shahuji Maharaj Medical University U.P., (formerly King George Medical University, Lucknow) India.*

**Keywords:** *Geriatric*, *Elderly*, *Antipsychotics*, *Antidepressants*, *Cholinesterase inhibitors*

## Abstract

With improvement in medical services in the last few years, there has been a constant rise in the geriatric population throughout the world, more so in the developing countries. The elderly are highly prone to develop psychiatric disorders, probably because of age related changes in the brain, concomitant physical disorders, as well as increased stress in later life. Psychiatric disorders in this population may have a different presentation than in other groups and some of psychopathologies might be mistaken for normal age related changes by an unwary clinician. Therefore the need of the day is to train psychiatrists and physicians to better recognize and manage mental disorders in this age group.

## Introduction

Standards of medical care have been improving for the last 50 or so years throughout the world. This has led to an increase in life expectancy of the population causing a surge in the size of the geriatric subgroup. By 1990, a clear majority (58%) of the world’s population aged 60 years and over was already to be found living in developing countries. By 2020 this proportion will have risen to 67%. Over this period of 30 years, this oldest sector of the population will have increased in number by 200% in developing countries as compared to 68% in the developed world (Murray & Lopez, 1996). Arrangements will have to be made to cater to the health needs of this ever-increasing subset of population.

According to the 2001 census, India is home to more than 76 million people aged 60 years and over. This age group, currently only 7.5% of the population, is expected to grow dramatically in the next few decades (projected to be 136 million by 2021). The percentage growth in the elderly population is almost double the rate of increase in the general population. Analysis of this data also highlights that the rate of demographic aging across India varies substantially. Regions with more favorable health indicators seem to be aging faster and the demand for specialist services will soon be evident in such places.

The elderly population is highly prone to develop psychiatric morbidities due to ageing of brain, problems with physical health, cerebral pathologies, socio-economic factors such as decrease in economic independence and breakdown of the family support system (Khandelwal, 2003).

Rates of mental disorder are much higher among elderly patients seen in primary care or hospitalized for medical conditions, 30–50% of whom have psychiatric conditions (Borson & Unutzer, 2000); and in long-term-care settings, 68–94% of residents have been found to have mental disorder (Hybels & Blazer, 2003).

The provision of safe drug therapy is considered one of the greatest challenges in the geriatric health care as patients in this subgroup, firstly, tend to suffer more commonly from multiple physical and psychiatric illnesses which may complicate each other’s course, secondly, on an average, the elderly tend to be taking more medications at any given time than other populations, which results in complicated interactions between the various drugs; thirdly, pharmacodynamic and pharmacokinetic changes with aging affect the way drugs and the body interact with each other.

There are various hurdles in the provision of good mental health care to the elderly (Young *et al*., 2001). These are:

- The presentation of psychiatric and physical illnesses may be different in the elderly as compared to the younger people. Physical disorders may distract the treating physician from psychiatric disorders, especially because of the time constraints of a standard brief office visit.
- Some psychiatric symptoms like memory disturbances, depression and anxiety might be considered part of a normal aging process, which they are not. Healthcare professionals with no training in psychiatry or geriatrics may find it difficult to distinguish signs of mental disorders from the normal aging process.
- Training in geriatric psychiatry is limited in India, and this limits the availability of quality mental health care for the elderly population.
- Many psychiatrists find it difficult to work with the elderly.

As has already been mentioned, there are various changes in the interaction between drugs and the body that occur with age. These are as follows:

1. Pharmacokinetic changes
2. Pharmacodynamic changes

## Pharmacokinetic Changes with Age

### Absorption

There is decrease in gastric acidity, delay in gastric emptying, decreased splanchnic blood flow and decreased intestinal motility with age. This results in slow absorption of drugs and a delay in their onset of action. [Desai, 2003, disagrees with this and states that all aspects of pharmacokinetics except absorption are affected by age].

### Metabolism

With age, there is decrease in the hepatic mass, decreased hepatic blood flow and decreased in certain methods of metabolism as compared to others. Hydroxylation and demethylation are examples of the latter, decrease in which leads to greater half-lives, prolonged action and accumulation of drugs such as diazepam and chlordiazepoxide (Vozeh, 1981; Wilkinson, 2007). However, glucuronide conjugation remains unaffected by age, making drugs such as lorazepam and oxazepam safer in the elderly (Greenblatt, 1981; Gareri *et al*., 2003).

P-450 CYP2D6 activity does not change with age but its inhibition by various psychiatric or non-psychiatric drugs can alter the effect of other psychotropics. Drugs like fluoxetine, paroxetine, venlafaxine, mirtazapine and valproate inhibit P450 CYP2D6, which affects metabolism of drugs like desipramine, nortriptyline (TCAs), paroxetine, venlafaxine, carbamazepine, risperidone, clozapine, olanzapine and typical antipsychotics. This may cause complications in treatment of psychiatric disorders which might involve co-administration of these medications.

### Excretion

There are various anatomical changes that take place in the renal system with advancement of age. There is a loss of renal mass, loss of glomeruli, thickening of the basement membrane of the glomeruli and the tubules, intimal thickening of the arterioles, obliteration of the arterioles in the cortical glomeruli (Davison, 1998; Clark, 2000). These changes result in reduced glomerular filtration rate (GFR). According to Berg (2006), there is approximately 8.7 ml/min/1.73m^2^ reduction in GFR per decade between the ages of 21–67 years. This reduction of the renal functions causes accumulation of drugs such as lithium and rivastigmine in the body even with normal dosages, resulting in drug toxicity.

### Distribution

Aging is usually accompanied by an increase in the percentage of body fat in the body resulting in an increase in elimination half-life of many psychotropic medications. There is also a decrease in the total body water with age resulting in decreased volume of distribution of lithium and increase in its concentration (Cusack *et al*., 1979; Andreucci *et al,*., 1996).

Less amount of plasma albumin in the elderly means a greater percentage of drug remains free in the blood which may result in higher incidence of adverse drug reactions. Decreased cerebral blood flow results in decreased availability of drugs to brain for action at a particular serum concentration.

## Pharmacodynamic Changes with Age

- The sensitivity of Gaba-aminergic system is known to increase with age which results in a heightened response to drugs such as benzodiazapines (*Greenblatt et al*., 1977).
- Due to age related decrease in the dopamine turnover, the elderly are at an increased risk of developing drug induce Parkinsonism (*Miguez et al*., 1999).
- Decreased cholinergic activity may result in an increased response to anticholinergic agents (*Umegaki et al*., 2000).
- Serotonin reuptake and 5-HT2A receptor concentration decreases with age (Druse *et al*., 1997).

### Major group of drugs used in the elderly patients:

1. Antipsychotic medications
2. Antidepressant medications
3. Mood stabilizers
4. Benzodiazapines and other anxiolytics
5. Anti-dementia drugs

### Antipsychotic medications

Psychoactive drugs remain the mainstay of treatment of psychosis in elderly, as in other population groups, although only a few studies investigating the effects of these drugs in the elderly were carried out (*Jeste et al*., 1996). Research is ongoing on the safe use of antipsychotic medications. In the meantime it is important not to do more harm than good and to simply follow the widely recommended principle of “start low, go slow” (Taylor *et al*., 2007).

Specific target symptoms must be identified before the start of antipsychotic medications and regular monitoring over the course of the illness needs to be done as exposing elderly patients to unwarranted medications may lead to various untoward effects. Even if the target symptoms have been identified, it is commonly seen that the elderly tend to experience a higher incidence of adverse drug reactions of antipsychotics as compared to their younger counterparts.

## Limitations of Typical Antipsychotics in the Elderly Population

### Anticholinergic side-effects

Central anticholinergic side-effects like poor attention, impaired memory and behavioral problems (Junginger *et al*., 1993; Lieberman, 2004) may lead to confusion regarding diagnosis and produce further deterioration in patients with pre-existing cognitive decline. Other anticholinergic side-effects such as urinary retention and constipation may also be particularly common in the elderly, especially in those who suffer from these conditions before the start of antipsychotic medications. It is therefore advised that the patients should be counseled regarding these side-effects beforehand. Once they develop, authors suggest that the dose of the medications should either be tapered down to the lowest effective dose or the patient switched to a different drug with lesser propensity to cause this problem. It is imperative that the treatment of pre-existing physical conditions (such as Benign Prostatic Hypertrophy) be given due importance.

### Extra-pyramidal side-effects (EPS)

As compared to the atypical antipsychotics, typical antipsychotics are associated with a higher incidence of extra-pyramidal dysfunction. Several studies have suggested that the geriatric population is more susceptible to neuroleptic-induced tremor than younger age groups (Caliguiri *et al*., 1999). Older patients with rigidity or tremor prior to medication treatment are most likely to develop neuroleptic-induced Parkinsonism. Hence, drugs with less likelihood of producing EPS (eg. Quetiapine) should be preferred for these individuals.

EPS are known to occur in as many as 80% of the elderly population receiving antipsychotic medications and may lead to difficulty in swallowing and falls (Tison *et al*., 1999). According to the author’s experience, certain steps can be taken to minimize the risk of falls in the elderly. These are, to start antipsychotic medications at a low dose and gradually increase it according to tolerability, maintaining adequate hydration, avoid using the stairs [if necessary, only with assistance], using low-lying beds, ensuring adequate lighting and constant vigilance by the attendants of the patient.

Akathisia due to neuroleptics may be mistaken for increased agitation in a patient with dementia and behaviour disturbance, which the clinician may erroneously respond to by raising, rather than lowering, the dose. Treatment with anticholinergic agents such as benztropine or trihexyphenidyl to ameliorate extrapyramidal symptoms is best avoided to reduce risk of anticholinergic toxicity, although low dosages (e.g., benztropine 0.5 mg twice daily) may be prescribed cautiously. Low-dose propranolol may improve akathisia, and amantadine may also help reduce rigidity, but these medications can also cause adverse effects.

Neuroleptic malignant syndrome (NMS) is a potentially life-threatening life condition with features of muscle rigidity, fever, autonomic instability, fluctuating levels of consciousness and elevations in CPK and leucocytes. NMS can be seen with the use of all antipsychotics, including the atypical ones (Johnson & Bruxner, 1998). NMS, of course, is more commonly seen in the young as compared to the elderly although old age disorders like dementia and Parkinsonism can predispose the person to developing it.

The risk factors for tardive dyskinesia (TD) in older people with schizophrenia include advancing age, the use of typical antipsychotics, duration of treatment, history of extrapyramidal symptoms or tremors prior to the neuroleptic treatment, female sex and organicity. Older patients may develop tardive movements after a shorter period of neuroleptic exposure than young patients, and the movement disorder remits less frequently after neuroleptic discontinuation. TD was found to be associated with increased psychopathology (positive and negative signs of schizophrenia) and Orofacial TD was associated with a lower MMSE score (cognitive impairment) in the study by Purandare *et al*, 2003. It is therefore recommended that patients who are on a long-term antipsychotic therapy should be screened regularly for TDs and decline in cognition (eg. once every six months).

### Cardiovascular side-effects

Orthostatic hypotension is a particular concern in elderly persons because of age-related limitations in vascular regulation and postural reflexes that can increase the risk of falls (hip fracture resulting from falls is a major cause of morbidity in older persons). The quinidine-like cardiac effects of typical antipsychotics, especially thioridazine, are associated with the potential for arrhythmias. These drugs should therefore be avoided in the elderly who are predisposed to developing arrhythmias.

### Other side-effects which may be seen in the elderly as a result of antipsychotic drug use are:

- Sedation
- Hypersalivation
- Gastrointestinal effects
  Nausea
  Constipation
  Diarrheaxy
- Liver effects
  Cholestatic jaundice
  Raised transaminase enzyme activities
- Endocrine effects
  Weight gain
  Diabetes mellitus
- Epilepsy

## Atypical Antipsychotics

### Clozapine

Clozapine continues to have a favourable neurological side-effect profile but its use in the elderly population is limited by its severe side-effects like sedation, confusion and agranulocytosis with resultant requirement of regular monitoring of blood counts. A few small studies on its use at lower doses in this population have reported sedation, lethargy and postural hypotension as common side-effects (reviewed by Barak *et al*., 1999). When prescribing clozapine, it is advisable to initiate treatment with a dose of 6.25 mg/day, followed by weekly titration of 6.25 mg/day until a therapeutic effect is achieved and/or side effects develop. Daily doses may range from 6.25 to 400 mg. (Sajatovic & Ramirez, 1995).

### Risperidone

Risperidone is effective in reducing aggressive behaviour as well as paranoia and delusions in elderly psychotic patients but it is known to induce EPS symptoms at low doses (1–2mg a day) in older patients, particularly those with pre-existing dementia. The recommended maximum dose of Risperidone in the elderly is 2–3mg/day.

### Olanzapine

Olanzapine appears to be more efficacious in maintaining control over negative symptoms in older patients with schizophrenia and related psychotic disorders in comparison to Risperidone. The optimally recommended dose range is 2.5–15 mg/day.

The most frequent adverse effects of Olanzapine are weight gain, hypotension, constipation, somnolence and dizziness (Stephenson & Pilowsky, 1999; Gomez *et al*., 2000). The side effect of weight gain may be of advantage in people who have suffered a significant weight loss during their illness.

### Quetiapine

Quetiapine is reported to inhibit 5-HT2 receptors more than D2 and affect mesolimbic pathways more than nigrostriatal ones which accounts for its efficacy in treating positive symptoms with a low propensity to cause EPS (McManus *et al*., 1999; Nagy, 2003). Common side-effects include sedation, headache, and orthostatic hypotension. Quetiapine was rarely associated with weight gain and it did not produce any substantial change in the thyroid and hematological profiles of the patients (McManus *et al*., 1999; Jaskiw *et al*., 2004). It is recommended that Quetiapine should be started with the lowest possible dose (25 mg) and slowly titrated up to 100–300 mg/day.

### Aripiprazole

Aripiprazole with its unique mode of action as a partial agonist at D2 receptors is less likely than the other atypicals to cause extrapyramidal symptoms, sedation, weight gain and cardiovascular side-effects (Hirose *et al*., 2004).

## Therapeutic Indications of Antipsychotics in Elderly Population

### Schizophrenia

The prevalence of schizophrenia [early, late onset (first symptom of schizophrenia develops after the age of 45 years) combine] in the population aged 65 years and above is believed to be about 1% (Cohen *et al*., 2000). Of these, most are people with early-onset schizophrenia who have reached old age while a minority are those who have developed schizophrenia in old age (Jeste & Twamley, 2003).

New onset of psychotic symptoms in an older person demands careful evaluation for medical, neurological, or pharmacological causes. Some evidence indicates that patients with late-onset schizophrenia may respond to lower neuroleptic dosages than those with early-onset schizophrenia who are now older (Spar & La Rue (a), 2006).

### Mood disorders with psychosis

Depression is the most common psychiatric disorder in the geriatric age group (Mayers *et al*., 1984; Meldon, 1997). Elderly depression is commonly associated with psychosis which may have the following effects on the course of the illness: increased risk of relapse, more persistent symptoms over 1 year, more attempts of suicide, greater number of hospitalizations and financial dependency (Lacro & Jeste, 1997). As outlined by Finn (2006), mania in the elderly is often found to have features such as irritability, confusion and paranoia (Yassa *et al*., 1988). Short-term treatment with antipsychotic medication may be beneficial, although antidepressant or mood-stabilizing pharmacotherapy is primary. Antipsychotic medication should generally be discontinued following remission of psychosis.

### Dementias with psychosis

Various studies have suggested that the use of antipsychotics for the control of psychotic symptoms in dementia is associated with various untoward side-effects such as an increased risk of mortality (Gill *et al*., 2007), stroke (Schneider *et al*., 2006), and falls (Landi *et al*., 2005) which led US FDA to issue a warning, first, on the use of atypical antipsychotics, and, later on, concerning the use of typical antipsychotics also in patients of dementia (U.S. Food and Drug Administration, 2005, 2008).

In spite of the above-mentioned risks, antipsychotic medications remain the mainstay of treatment of psychosis in dementia, as anti-dementia drugs such as donepezil are not effective in controlling these symptoms, especially agitation (Howard *et al*., 2007).

Psychosis in Parkinson’s disease merits a special mention because, firstly, drugs used in the treatment of this disorder (levodopa, amantadine, monoamine oxidase inhibitors) are known to cause psychosis, and secondly, people with parkinson’s disorder are at an increased risk of developing EPS in response to both typical as well as atypical antipsychotic medications. It is recommended that before start of antipsychotic medications, anti-parkinsonian medications must be brought down to a minimum possible level, just enough to control the movement disorder. If this does not work, antipsychotic medication should be started. Studies have shown efficacy of clozapine in low doses, up to 50mg per day (Hoeh *et al*., 2003), in treating psychosis with Parkinson’s disease; but due to its adverse drug profile, it is not commonly used. Use of Risperidone and Olanzapine even in low doses has been shown to be associated with increase in tremor and rigidity (Breier *et al*., 2002). Quetiapine may be best tolerated in this population but there has been limited clinical experience (Weintraub *et al*., 2007). Dose of 25-300 mg/day is probably optimal for most patients (Fernandez *et al*., 1999; Weintraub *et al*., 2007).

### Antidepressants

As has already been pointed out, the elderly are known to have more stressors in their lives like ageing of brain, problems with physical health, cerebral pathologies, socio-economic factors such as decrease in economic independence and breakdown of the family support system. These problems have been found to be associated with the development of depression in the elderly. The elderly are also prone to suffer from serious consequences of depression like deterioration of health due to decreased intake of food, decreased self-care, and loss of adherence to medications for other physical disorders such as diabetes and hypertension, which may lead to serious repercussions. Suicidal attempts in the elderly are known to be more lethal than in the younger population.

Medications used to treat depression in the elderly:

- Selective serotonin reuptake inhibitors (SSRIs) (e.g. fluoxetine, sertraline, paroxetine, fluvoxamine, citalopram, and escitalopram)
- Non-Selective Serotonin Reuptake Inhibitors (bupropion, venlafaxine, and mirtazapine)
- Tri-cyclic antidepressants (TCA) (e.g. amitriptyline, imipramine, doxepin, trimipramine, desipramine, nortriptyline and protriptyline)

### SSRIs

SSRIs are now the drug of choice for elderly depression because of their efficacy and favourable side effect profile and safety in overdose (Rivard, 1997; Spar & La Rue (2006b). The side-effects of SSRIs, that are expected to improve with continued use, are mild anorexia, nausea, gastrointestinal upset, jitteriness, and headache, while others, such as sexual dysfunction and weight gain, may not improve with time. The patient should be counseled beforehand regarding these side-effects. The choice of medication may depend on the distress that these side-effects will produce in the patients. Patients who are not sexually active might not be bothered by these side-effects whereas those who are might find them stressful. If required, the prescription can be changed to a drug with less propensity to cause sexual dysfunction, such as bupropion.

All SSRIs competitively inhibit several cytochrome P450 (CYP) isoenzymes and have significant interaction with many other drugs such as other antidepressants, antipsychotics, ACE inhibitors, opioids and beta-blockers (increased levels) and benzodiazapines, hypnotics (zolpidem), calcium channel blockers and anti-histaminics (decrease levels) (Nemeroff *et al*., 1996; Hemeryck & Belpaire, 2002). Partoxetine is considered to be the one with the highest drug interactions. In this regard, escitalopram and sertraline are known to have lesser interactions with other drugs and the former is considered the anti-depressant of choice in patients receiving complex multi-drug regimens (Solai *et al*, 1997; Hemeryck & Belpaire, 2002, Ravindran *et al*., 2005). The starting and maintenance doses of SSRIs in the geriatric population are given in the Table 1.

**Table 1:** Antidepressants in geriatric population

Drug	Starting dose	Usual maintenance dose
Paroxetine	10-20 mg/day	20-30 mg/day
Sertraline	25-50 mg/day	50-100 mg/day
Escitalopram	05-10 mg/day	10-20 mg/day
Fluoxetine	10 mg/day	20 mg/day

Note: Table based on the clinical experience of co-author (J.K.T.).

### Non-selective serotonin reuptake inhibitors

- Bupropion- The dopaminergic properties of bupropion may be beneficial for patients with Parkinson’s disease. It is very safe in overdose and it does not cause weight gain (Settle *et al*., 1999; Croft *et al*., 2002). However, it may increase blood levels of certain anti-psychotic medications,β-blockers, and type 1C antiarrhythmic agents by inhibiting the CYP2C6 isoenzyme.
- Venlafaxine- Side-effects such as sexual dysfunctions, weight gain, gastro-intestinal disturbances are fairly commonly seen with it. Venlafaxine is also known to increase the supine blood pressure in 3–5% of patients. Hence monitoring of BP should be done. Downward adjustment of dosage is recommended in patients with liver or kidney disease.
- Mirtazapine- It is a safe antidepressant with sedating properties. It is associated with increased appetite and weight gain, which may be of use in patients of depression with reduced intake of food.

### TCAs

Tertiary tricyclics (e.g., amitriptyline, imipramine, doxepin, trimipramine) are not considered safe in the elderly population because of their greater sedating, anticholinergic, and cardiac side effects and their relatively greater tendency to cause orthostatic hypotension. However, their demethylated counterparts, the secondary tricyclics (e.g., desipramine, nortriptyline, protriptyline) have a lower propensity to cause the above-mentioned side-effects and are therefore considered safe in the geriatric population (Salzman & DuRand, 1994; Alvarez & Pickworth, 2003). All TCAs have type 1 anti-arrhythmic effects (Roose & Glassman, 1994; Alvarez & Pickworth, 2003) and are relatively contraindicated in patients with ischemic heart disease or preexisting cardiac conduction disturbance (increased P-R interval, bundle-branch block, increased QRS).

TCAs are not preferred in the treatment of bipolar depression as they are associated with switching from depression to hypomania or mania.

## Mood Stabilizers

### Lithium

As the elderly have a lower renal function as compared to the younger population, they suffer from side-effects of lithium therapy like diarrhea, tremor, polyurea and polydipsia more commonly (Smith & Helms, 1982; Mohandas & Rajmohan, 2007). Delirium, sedation, and “cognitive dulling” can occur but are typically seen at higher serum levels (i.e., >1.0 mEq/mL). Side-effects of long-term lithium therapy may include hypothyroidism and goiter, and the question of renal tubular damage remains controversial (Abou-Saleh, 2002).

It was seen that people who earlier tolerated lithium well might be unable to do so after the age of about 70 years. McDonald and Nemeroff, 1998, advised that using sustained release preparation which decreases the peak levels, or shifting the medication to night time so that the primary side-effects occur when the patient is asleep, might help to improve the tolerability of lithium in the said population. In the elderly, with their decreased muscle mass, measuring serum creatinine to test the renal function can be misleading. According to Gareri *et al*. 2003, a better test of renal function and lithium excretion in the elderly would be measuring creatinine clearance, preferably by collecting a 24hr urine sample, although using the following formula can be a faster method of determining the same:

Creatinine clearance = (140 - age in years) × bodyweight (kg)/72 × plasma creatinine (mg/dL).

As lithium therapy is associated with these untoward effects, it is essential that a pre-treatment physical and laboratory workup of the patient should be done prior to the commencement of lithium therapy. This pre-lithium workup should include the following:

- Thorough physical examination to detect thyroid enlargement, tremor, signs of renal dysfunction (exacerbated by lithium), and congestive heart failure (use of diuretics may increase the levels of lithium).
- Electrocardiogram should be done to rule out sick-sinus syndrome, which is a relative contraindication for lithium therapy, as bradycardia and arrhythmias may be precipitated. Benign nonspecific T-wave abnormalities and U waves that reverse with cessation of treatment are common in patients taking lithium and do not require discontinuation of treatment.
- Laboratory investigations are meant to detect renal dysfunctions (serum urea, serum creatinine, serum electrolytes) and thyroid disorders (T-3, T-4 and TSH). Re-evaluation of thyroid functions should be done at regular intervals in patients receiving lithium therapy.

It is important to remember that lithium levels and half life are increased in the elderly due to decrease in the GFR because of which the prescribed dose of lithium in elderly should be less than that prescribed in the young. Plasma levels in the range of 0.3–0.8 mEq/mL should be aimed at for maintenance therapy (Abou-Saleh, 2002).

Another important point to keep in mind regarding the use of lithium in elderly is that most people in this population take drugs such as NSAIDS, thiazide diuretics and ACE inhibitors which increase the levels of lithium and cause drug toxicity.

### Valproate

Valproate is considered an acceptable alternative to lithium in manic elderly patients, particularly in those who develop deterioration of cognitive performance during lithium treatment (Young *et al*., 2004). It is the drug of choice for rapid cycling bipolar disorder. There is no study to our knowledge which mentions that the drug of choice is different in the young and the elderly.

Dosages of Valproate in the elderly should be adjusted to attain plasma levels of around 50 and 120μg/mL. Prescribing 400-1000mg of Valproate per day in divided doses usually produces the desired concentration in blood. The dosage should be kept between 400-1000mg per day to produce a serum concentration of 50-120mcg/ml. Here too, no study, to our knowledge, gives separate doses for the young and the elderly. Side effects with this dosage are usually minimal and include sedation, nausea, and ataxia. However, it is still recommended that the dosage of valproate in elderly should be increased slowly as compared to the young along with regular monitoring (Barclay, 2002).

### Carbamazepine

Rapid cycling and occurrence of irritable mania are features known to occur relatively more commonly in the elderly population. Carbamazepine is effective in managing both these symptoms (Greil *et al*., 1998, Weisler *et al*., 2005). Dosages of carbamazepine (typically beginning at 200 mg orally twice a day) are adjusted to produce blood levels in the range of 4–12 ng/mL, and antimanic effects appear after four to seven days. Dosage increase is commonly required after four to six weeks of therapy to maintain therapeutic blood levels because carbamazepine induces its own metabolism.

Pre-treatment investigations in case of Carbamazepine should include the total blood picture as rare cases of aplastic anaemia and agranulocytosis have been reported. Other side-effects of Carbamazepine therapy include sedation, dizziness, ataxia, nausea and vomiting, mild anticholinergic effects, skin rash (rarely including Stevens-Johnson syndrome and toxic epidermal necrolysis), and worsening of congestive heart failure, hypertension, and hypotension (Ladefoged & Mogelvang, 1982, Croft, 2009).

### Lamotrigine

A study conducted by Robillard and Conn in 2002 revealed that lamotrigine added to lithium or divalproex led to remission (Robillard & Conn, 2002). To reduce the risk of rash, a rigid schedule of dosage increases is required: for nonelderly adults, it is 25 mg once daily for one week, 50 mg once daily for the second week, and 100 mg once daily for the last 2 weeks. Elderly patients may require even smaller dosages at each step which should be based upon the tolerability of the patient for lamotrigine.

## Benzodiazepines and Other Anxiolytics

Benzodiazepines remain the treatment of choice for anxiety of acute or subacute duration. For anxiety disorders requiring treatment for more than 4–6 weeks, shorter-acting benzodiazepines such as lorazepam or oxazepam, which undergo single-step conjugation in the liver and have no active metabolites, are generally preferable. Longer acting benzodiazepines tend to accumulate in the body when used for such a long period and cause untoward side-effects. Lorazepam and oxazepam have the additional advantage of being relatively well tolerated by patients with even fairly advanced liver disease. The efficacy of benzodiazepines in treatment of anxiety in young as well as the elderly diminishes beyond four to six weeks and other agents such as antidepressants need to be added to produce an anxiolytic effect.

Buspirone is another nonbenzodiazepine that has been shown to be an effective anxiolytic agent in elderly patients (Goldberg, 1994; Cassidy & Rector, 2008).

Advantages of buspirone over benzodiazepines are:

- No sedative effects,
- No interaction with alcohol,
- No tendency to produce dependence or withdrawal symptoms; therefore, buspirone has little or no abuse potential.

The disadvantages in the use of buspirone as an anxiolytic are:

- Usually several weeks of daily dosage are required before effects are observed.
- Buspirone is contraindicated in patients taking mono-amine oxidase inhibitors (presumably because of its serotonergic properties)
- Less effective in patients who have been treated with benzodiazepines (Schwizer *et al*., 1986).

Zolpidem is amongst the most widely used hypnotic in the elderly (Shaw *et al*., 1992) and zaleplon is much shorter acting and as effective as zolpidem. Recently a newer molecule eszopiclone has been proven to be an efficacious hypnotic with bitter taste being its most common side effect (Mack & Salazar, 2003). These non-benzodiazepine hypnotics have low abuse potential and low rate of exacerbation of sleep apnea.

## Anti-dementia Drugs

### Cholinesterase inhibitors

Several cholinesterase inhibitors (tacrine, donepezil, rivastigmine, and galantamine) are available for treatment of the cognitive deficits of mild to moderate Alzheimer’s disease. All of these agents increase central cholinergic neurotransmission by inhibiting breakdown of acetylcholine by acetylcholinesterase.

Tacrine- it is known to be hepatotoxic. Use is not recommended.

Donepezil- most commonly used cholinesterase inhibitor. The main side-effects of donepezil include nausea, diarrhoea and insomnia. According to Benazzi and Rossi, 1999, donepezil may help in depression and can exacerbate mania. Dose: 10 mg/day.

Rivastigmine- more centrally acting cholinesterase inhibitor and is considered safe for hepatic function. The usual dose is 6–12 mg/day.

Galantamine- it is a newer acetylcholine esterase inhibitor. Main side-effects of galantamine are nausea, vomiting, diarrhea, anorexia, weight loss, abdominal pain, dizziness and tremors (more frequent early in the course of treatment and during dose titration). The recommended dose is 6–12 mg/day.

One 36-month follow-up study found that almost 80% fewer cholinesterase inhibitor-treated subjects were admitted to nursing homes compared with untreated subjects (Lopez *et al*., 2005).

### Memantine

Memantine is the first noncholinesterase inhibitor that has been shown in randomized controlled clinical trials, to be effective in reversing or slowing cognitive and functional decline in Alzheimer’s disease (Areosa *et al*. 2005). The drug is an antagonist of the N-methyl-D-aspartate (NMDA)–type glutamate receptor and appears to exert its effects at the cellular level by reducing glutamate mediated cytotoxicity, which leads to neuronal injury or death. Unlike the cholinesterase inhibitors, memantine is effective in patients with moderate to severe Alzheimer’s disease and seems to exert atleast additive effects when combined with cholinesterase inhibitors (Tariot *et al*. 2004).

Improvement in agitation and mood has been observed in several clinical trials, but cognitive and functional decline in Alzheimer’s disease remains the main indication. The initial dose is 5 mg/day, which is increased in 5-mg/day increments over four weeks to the final dose of 10 mg twice per day. Side effects are minimal, with dizziness, confusion, headache, and constipation being most often reported. Memantine undergoes little metabolism, and adverse drug-drug interactions have not been reported.

### Other drugs

Sano *et al*., (1997) reported that patients with Alzheimer’s disease who were taking 2,000 IU of vitamin E showed no improvement in cognitive function but did take significantly longer to require institutionalization than did those receiving either placebo or 10 mg/day of selegiline. Although this finding has not been replicated, it has stimulated some clinicians to add high-dose vitamin E to the treatment regimen of patients with Alzheimer’s disease.

Drugs like NSAIDS, hormones, statins, and ginkgo biloba have been studied in patients with Alzheimer’s disease but a definitive evidence of their efficacy is still not available.

## Practical Points to Keep in Mind

- Keep the regimen as simple as possible.
- It is important not to do more harm than good and to simply follow the widely recommended principle of “start low, go slow”.
- Maintenance dose should be about 1/3-1/2 of average adult dose although some elderly patients may require full dose of the medication.
- Try not to treat the side-effects of one drug with another drug. Find a better tolerated alternative.
- Time-limit the prescriptions so that ineffective treatment is not unnecessarily continued.
- Avoid under treatment, for example of depression and cognitive impairment. Do not neglect depressed mood and impaired memory by considering them a normal state of aging.
- Maintain an optimistic and supportive attitude.

## Conclusion

According to Murray and Lopez (1996), the percentage of geriatric population will increase by 200% and 68% in developing and developed countries respectively, from 1990 to 2020. According to the 2001 census of India, about 7.5% of the population is above 60 years of age and it is expected to increase with time. Physicians and psychiatrists trained in the recognition and management of both physical and mental disorders need to cater to the health needs of this ever-increasing part of the population. Due to age related changes in the body, the elderly are predisposed to develop adverse drug reactions. Drug studies with older population as their subjects are lacking; hence there is a need for further research, studies which focus on various co-morbidities in the geriatric population.

**Figure 1:**
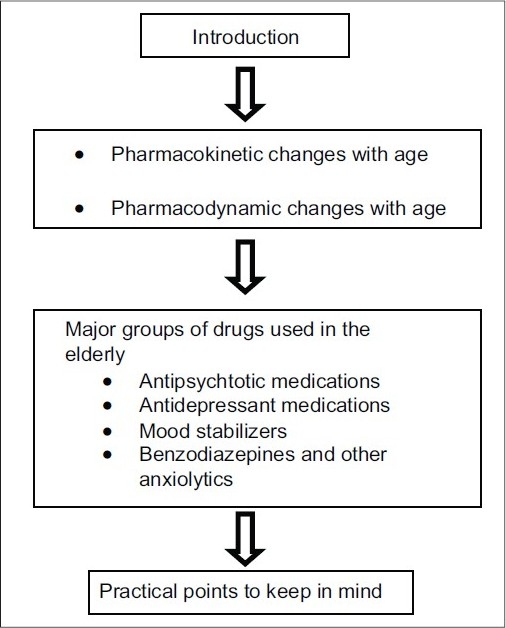
Flow chart of the paper

### Take home message

The elderly population is at a constant rise and there is an urgent need to meet their mental and physical health requirements. Psychiatric disorders may present with different features and follow a more complicated course in the geriatric population because of age related changes in the body as well as comorbid conditions (including medications for these disorders). While managing psychiatric disorders in the elderly, these factors must be kept in mind.

### Conflict of interest

The authors declare no conflicting interests in relation to the content or preparation of this manuscript.

### Declaration

This is to confirm that this is our original unpublished piece, not submitted for publication elsewhere.

### Principal and corresponding authors

This is to certify that there is no conflict of interest of any kind, regarding the paper amongst the authors.

## Questions That This Paper Raises

1. What further research is required in the area of safety and efficacy of psychotropic medications in geriatric population?
2. What is the research required in the area of liaison psychiatry to better understand geriatric psychiatric disorders as comorbid conditions (presentation of symptoms and their effective management)?
3. What further steps can be undertaken to improve learning and training of psychiatrists in this specialized area and at what level (undergraduate training/ part of post graduation training/ specialized courses)?
4. How can the general medical practitioners be taught to better recognize the signs and symptoms of geriatric psychiatric disorders so that early detection and management can be imparted to these patients?
5. What further research is needed in the field of mild cognitive impairment and the factors which might prevent or cause its progression into dementia?

## About the Author


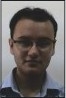
 *Dr. Sannidhya Varma is currently pursuing his Master’s degree in Psychiatry from Department of Psychiatry, CSM Medical University, Lucknow, India. He has done his MBBS from Government Medical College and Hospital, Chandigarh, and has been a meritorious student throughout. He is academically and clinically oriented and has been an active participant in various zonal and national conferences. During his tenure of residency, he has got training in the fields of general adult, child and adolescent, geriatric, sex clinic and de-addiction psychiatry. E-mail: sannidhya.varma@yahoo.com, sannidhya.varma@gmail.com*
